# Non-Human Primate Model of Kaposi's Sarcoma-Associated Herpesvirus Infection

**DOI:** 10.1371/journal.ppat.1000606

**Published:** 2009-10-02

**Authors:** Heesoon Chang, Lynn M. Wachtman, Christine B. Pearson, Jong-Soo Lee, Hye-Ra Lee, Steven H. Lee, Jeffrey Vieira, Keith G. Mansfield, Jae U. Jung

**Affiliations:** 1 Department of Molecular Microbiology and Immunology, University of Southern California, Keck School of Medicine, Los Angeles, California, United States of America; 2 Department of Microbiology and Molecular Genetics and Tumor Virology Division, New England Primate Research Center, Harvard Medical School, Southborough, Massachusetts, United States of America; 3 Primate Medicine Division, New England Primate Research Center, Harvard Medical School, Southborough, Massachusetts, United States of America; 4 Department of Laboratory Medicine, University of Washington, Seattle, Washington, United States of America; Oregon Health & Science University, United States of America

## Abstract

Since Kaposi's sarcoma-associated herpesvirus (KSHV or *human herpesvirus 8*) was first identified in Kaposi's sarcoma (KS) lesions of HIV-infected individuals with AIDS, the basic biological understanding of KSHV has progressed remarkably. However, the absence of a proper animal model for KSHV continues to impede direct *in vivo* studies of viral replication, persistence, and pathogenesis. In response to this need for an animal model of KSHV infection, we have explored whether common marmosets can be experimentally infected with human KSHV. Here, we report the successful zoonotic transmission of KSHV into common marmosets (*Callithrix jacchus*, Cj), a New World primate. Marmosets infected with recombinant KSHV rapidly seroconverted and maintained a vigorous anti-KSHV antibody response. KSHV DNA and latent nuclear antigen (LANA) were readily detected in the peripheral blood mononuclear cells (PBMCs) and various tissues of infected marmosets. Remarkably, one orally infected marmoset developed a KS-like skin lesion with the characteristic infiltration of leukocytes by spindle cells positive for KSHV DNA and proteins. These results demonstrate that human KSHV infects common marmosets, establishes an efficient persistent infection, and occasionally leads to a KS-like skin lesion. This is the first animal model to significantly elaborate the important aspects of KSHV infection in humans and will aid in the future design of vaccines against KSHV and anti-viral therapies targeting KSHV coinfected tumor cells.

## Author Summary

Kaposi's sarcoma-associated herpesvirus (KSHV or *human herpesvirus 8*), the most recently identified human tumor-inducing virus, has been linked to Kaposi's sarcoma, pleural effusion lymphomas and multicentric Castleman's disease. In fact, KSHV accounts for a large proportion of the cancer deaths in Africa. Further, the incidence of KSHV in the US and Europe has greatly increased due to the AIDS pandemic. Despite these pressing human health problems, studies of KSHV infection are greatly hampered by the lack of cell culture and animal models. To address this serious need, we set out to develop an animal model for KSHV infection. In this manuscript, we report the successful zoonotic transmission of KSHV into common marmosets (*Callithrix jacchus*, Cj), a New World primate. Our study demonstrates that experimental KSHV infection of the common marmoset is highly analogous to its infection of humans, including the means of infection, sustained serological responses, latent infection of PBMCs, virus persistence, and KS-like skin lesion development, although the latter was infrequent in experimental KSHV infections. This model thus provides a unique opportunity to dissect the molecular mechanisms of KSHV infection, persistence, and pathogenesis directly in primates.

## Introduction

The most recently described human tumor virus, KSHV is a γ-2 herpesvirus and was first identified in association with KS, the most common neoplasm amongst AIDS patients [1]. KS is clinically separated into four different forms: classical KS, endemic KS, iatrogenic KS, and epidemic HIV-associated KS [2]. In addition to KS, KSHV is linked with two other cancers, Primary effusion lymphoma (PEL) [1],[3],[4] and Multicentric Castleman's disease [5],[6],[7]. Both of these cancers are B cell proliferative disorders, generally have poor outcomes, and have short median survival times complicated by their association with AIDS.

One experimental barrier to working with KSHV has been the lack of an *in vitro* system for examining lytic replication. While KSHV can infect a wide variety of primary cells and cell lines, none support the growth of KSHV to a high titer [8]. Typically, viruses can be stimulated toward replication only through the addition of agents like phorbol esters [9], this limitation extending to the *in vivo* setting. These problems had previously been addressed in two ways: through manipulation of the virus for increased titer or cell infectivity and the use of highly related viruses. By inserting a gene conferring resistance to an antibiotic, one can select cell populations that are essentially 100% infected [10]. Meanwhile, two examples of related viruses used as stand-ins for KSHV are Herpesvirus saimiri (HVS) [11] and Rhesus rhadinovirus (RRV) [12],[13]. These viruses are largely co-linear with KSHV, carry many of the same genes, and are known to infect non-human primates [13]. RRV infection develops abnormal cellular proliferations characterized as extranodal lymphoma and retroperitoneal fibromatosis, a proliferative mesenchymal proliferative lesion, in an experimentally co-infected rhesus macaque with simian immunodeficiency virus, suggesting an excellent primate model to investigate KSHV-like pathogenesis [14],[15]. In the case of HVS, infection of New World primates results in an aggressive, fulminant lymphoma. However, HVS primarily infects T cells, not B cells, as KSHV does. RRV persists upon infection in rhesus macaques, infects B cells, and induces B cell hyperplasia, but no KS-like disease occurs [15]. On the other hand, murine Herpesvirus 68 (MHV-68) provides a small, experimentally accessible mouse model, but its infection does not associate with KS or related diseases [16]. The introduction of KSHV genes into these systems has proven to be useful, albeit limited, for the study of KSHV [17].

Besides these related virus models, *in vitro* experiments and transgenic animal models have been the main forces in elucidating the potential roles of individual KSHV proteins in cell culture and mouse models, respectively [18],[19],[20],[21]. In a recent study, SCID-hu Thy/Liv mice reconstituted with the liver and thymus of human fetuses were utilized to study viral transcription as well as the susceptibility of the mice to infection with BCBL-1 derived KSHV [19],[22]. In addition, Parsons et. al have shown that NOD/SCID mice infected with purified KSHV provide a system for demonstrating latent and lytic viral gene expression in addition to cell tropism [19],[22]. Furthermore, they have investigated immune responses to KSHV via implanted NOD/SCID mice reconstituted with human fetal bone, thymus, and skin [19],[22]. In spite of these significant improvements, none of these models truly reflect the *in vivo* setting. To understand the relative contributions of KSHV proteins to the cellular activation of KSHV-associated diseases and host-viral interactions for viral persistent infection, an animal model that provides a complete viral infection in addition to latent and lytic viral gene expression within the context of an intact host immunity still needs to be developed. In this report, we describe the efficient zoonotic transmission of KSHV into common marmosets (*Callithrix jacchus*, Cj), a New World primate. Common marmosets intravenously inoculated with recombinant KSHV rapidly seroconverted and maintained high antibody responses for over one and a half years. In addition, KSHV DNA and LANA proteins were readily detectable in PBMCs and various tissues of the infected marmosets at a variety of time points. Furthermore, two common marmosets inoculated with rKSHV.219 by the oral route seroconverted and were positive for viral DNA in their PBMCs. Remarkably, a common marmoset infected with rKSHV.219 via the oral route developed a KS-like lesion with the characteristic spindle cells along with small blood vessels and extravasated erythrocytes. These results demonstrate that human KSHV effectively infects common marmosets, establishes persistence, and occasionally associates with the development of KS-like skin lesions. This is the first animal model of KSHV persistent infection to allow for analyses of the molecular mechanisms of the KSHV lifecycle directly in a non-human primate.

## Results/Discussion

### Experimental infection of common marmosets with rKSHV.219

**Figure 1:**
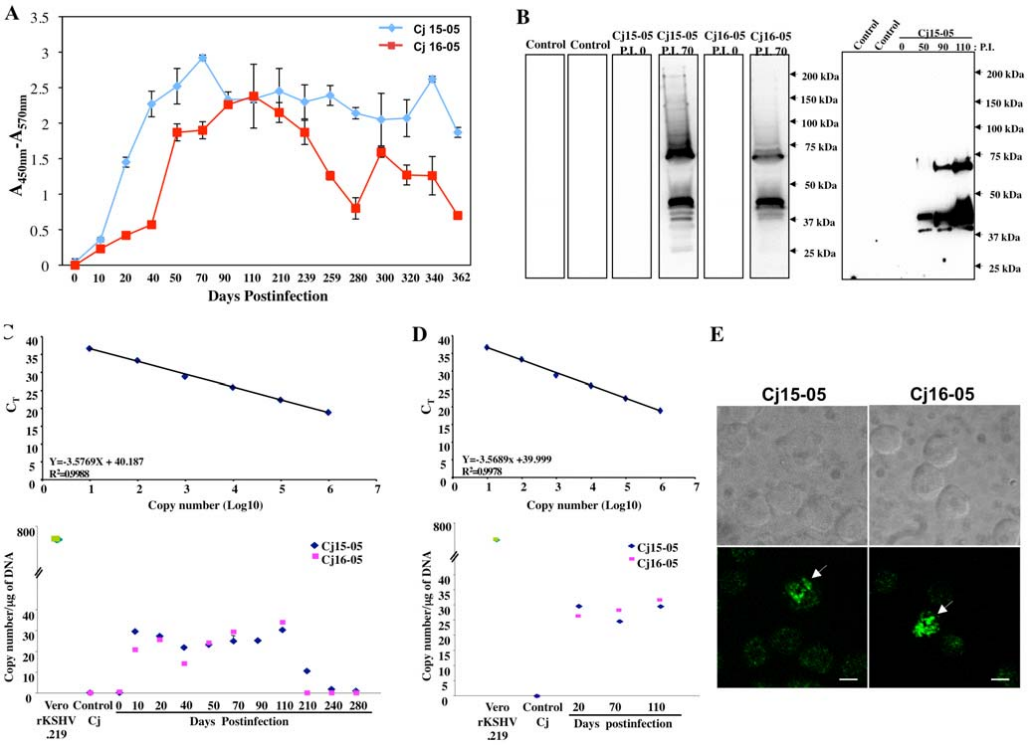
Experimental infection of common marmosets with rKSHV.219. (A) ELISA using the sera (1∶100 dilution) of infected marmosets (Cj15-05 and Cj16-05) against the lysed recombinant virus. The results show the averages of four repeats and standard deviations. These assays were reproduced by at least four independent occasions. (B) Immunoblot using the sera of infected marmosets (Cj15-05 and Cj16-05). Sera (1∶500 dilution) taken from naïve control marmosets (Control) and infected marmosets (Cj15-05 and Cj16-05 in the left panel and Cj15-05 in the right panel) at various time points were used to immunoblot 20 µg of purified virion proteins. P.I., days postinfection. (C and D) Real-time PCR analyses of KSHV LANA and ORF9 DNA. Purified genomic DNA (100 ng) from the PBMCs of three naïve controls and two infected (Cj15-05 and Cj16-05) marmosets was used for real time-PCR with primers specific for LANA (c) and ORF9 (D). Purified genomic DNA (100 ng) from Vero.rKSHV.219 cells was included as a control (P) in (C). DNA copy number was calculated using a standard curve with pcDNA3-ORF9 DNA and presented as the viral copy number per µg of DNA. M, 100-bp DNA molecular markers. The results show the averages of four repeats and standard deviations. (E) Detection of the LANA protein by confocal microscopy. The PBMCs of KSHV-infected marmosets were subjected to immunostaining with anti-LANA, followed by confocal microscopy. The arrows indicate LANA-positive cells. The top panels show phase contrast images of the PBMCs. The scale bars represent 20 µm. It should be noted that this picture shows an example of a rare positive field, but not reflecting the overall incidence of LANA positive cells.

Given its magnitude as a human health problem, it is crucial to understand the molecular details of KSHV biology. However, the lack of an animal model of KSHV infection greatly hampers studies of KSHV pathogenesis and persistence. Therefore, we explored whether common marmosets can be experimentally infected with human KSHV, and if so, to what extent experimental infection recapitulates the important aspects of KSHV infection. To facilitate the infection of common marmosets with KSHV, we chose a recombinant KSHV, rKSHV.219, from KSHV-infected JSC-1 cells [23]. rKSHV.219 expresses red fluorescent protein (RFP) from the KSHV lytic PAN promoter, green fluorescent protein (GFP) from the EF-1α promoter, and contains the gene for puromycin resistance as a selectable marker [10]. Two common marmosets (Cj15-05 and Cj16-05) were inoculated intravenously with 5×10^6^ infectious units (IU) of rKSHV.219. Blood samples were obtained at various time points to measure the marmosets' antibody responses and to detect viral DNA and proteins. Both monkeys quickly seroconverted to anti-KSHV-positive status within 20 days after inoculation (Fig 1A) and the animals' anti-KSHV antibodies persisted at very high levels for over 1.5 years. In addition, the sera from both infected monkeys readily reacted with purified KSHV virion proteins on immunoblots, with higher antibody reactivities detected over time (Fig 1B). A positive control, immunoreactive serum from a KSHV-infected patient was included to validate this immunoblot assay (Supplemental Fig S1). PBMCs from KSHV-infected animals were PCR-positive for KSHV LANA and ORF9 10 and 20 days after infection, respectively (Fig 1C and 1D). Viral DNA persisted in the PBMCs of monkey Cj15-05 for the entire 1.5- year span the animal was studied, whereas the level of viral DNA in monkey Cj16-05 decreased after 200 days postinfection (Fig 1C). Real-time PCR analysis indicated that the KSHV DNA copy number/µg of genomic PBMC DNA from the infected monkeys were substantially lower than that of rKSHV.219-infected Vero cells (Fig 1D). The low viral DNA copy number suggests that a minor population of the monkey PBMCs carried the rKSHV.219 genome. This was confirmed by confocal microscopy which showed that the KSHV LANA protein was detected in monkey PBMCs at a frequency of 2–5 cells per 1×10^6^ cells (Fig 1E). It should be noted that this figure depicts a rare positive field and does not reflect the overall incidence of LANA-positive cells. However, the infection frequency is similar to that of other γ-2 Herpesviruses, such as Rhesus lymphocryptovirus, Rhesus rhadinovirus, and Herpesvirus saimiri, which persist asymptomatically in their natural hosts [11],[12],[15],[24]. Due to the lack of an efficient *in vitro* culture system for KSHV infection and replication, virus recovery from the PBMCs of the experimentally infected marmosets was unsuccessful. Nevertheless, these results unambiguously demonstrate the persistent infection of naïve common marmosets by rKSHV.219.

**Figure 2:**
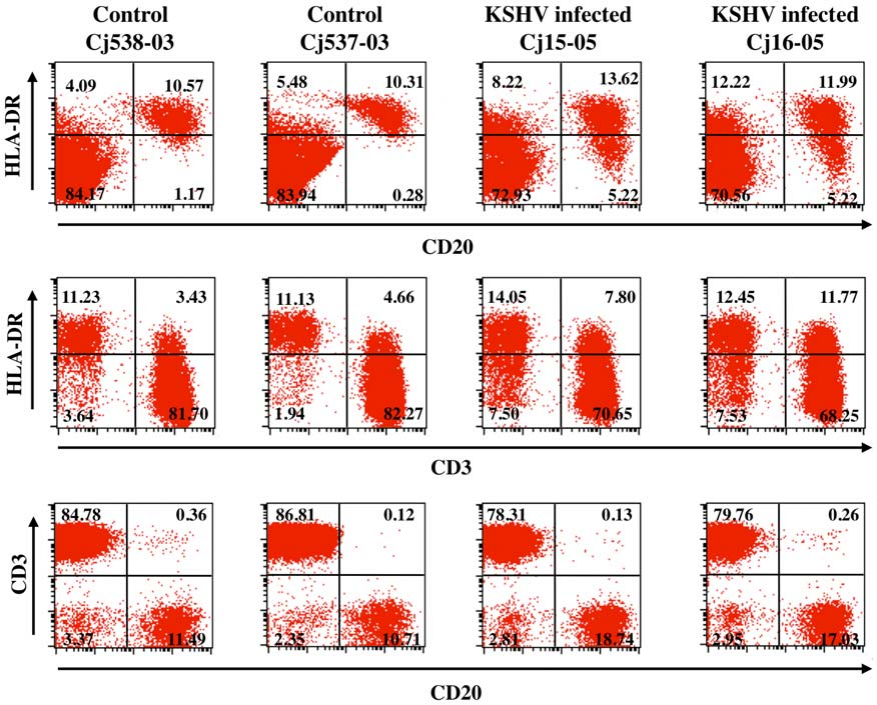
Increase in CD20^+^ B cell populations in common marmosets infected with rKSHV.219. PBMCs from Cj15-05 and Cj16-05 at day 210 P.I. were used for flow cytometry analysis with anti-CD3, anti-CD20, and anti-HLA-DR to identify T cells, B cells, and activated lymphocytes, respectively. The PBMCs from uninfected marmosets Cj538-03 and Cj537-03 were used as controls. The numbers in the boxes indicate percentages relative to the entire PBMC population.

Analysis of CD20^+^ B cells in the blood of the two infected marmosets showed increased B cell populations when compared to naïve marmosets (Fig 2). Although the total number of B cells did not change significantly in the initial few months after infection with rKSHV.219, their numbers noticeably increased around seven months postinfection and remained relatively elevated for as long as we followed these animals. Naïve common marmosets had 10–15% CD20^+^ B cells in their PBMCs while rKSHV.219-infected marmosets Cj15-05 and Cj16-05 had 15–20% CD20^+^ B cells (Fig 2 and Supplemental Fig S2). Interestingly, a 7 fold increase in HLA-DR^−^ CD20^+^ B cells at 200 days postinfection was observed in marmosets infected with rKSHV.219 (Fig 1F) and this population was maintained until these animals were euthanized (data not shown), indicating that rKSHV.219 infection leads to elevated levels of CD20^+^ B cells in marmosets. However, no B cell hyperplasia was observed in either monkey (data not shown).

### Early infection of rKSHV.219 in common marmoset

**Figure 3:**
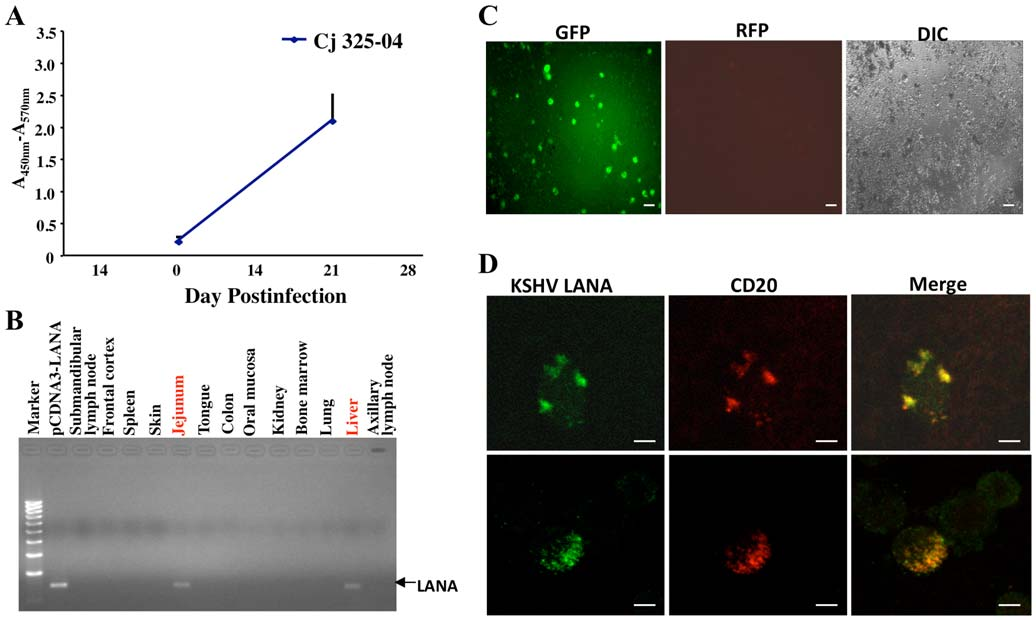
The early infection of rKSHV.219 in common marmoset. (A) Anti-KSHV ELISA with sera from Cj325-04 (1∶100 dilution) was performed as described in Figure 1A. The results show the averages of two repeats and standard deviations. These assays were reproduced by three independent occasions. (B) The PCR products of KSHV DNA from various tissues of infected marmoset Cj325-04 at day 21 P.I. (C) Immunofluorescence and differential interference contrast microscopy [34] of PBMCs from Cj325-04 at day 21 P.I. The scale bars represent 30 µm. It should be noted that this picture shows an example of a rare positive field, but not reflecting the overall incidence of GFP-positive cells. (D) The PBMCs of the KSHV-infected marmoset (day 21 P.I.) were subjected to immunostaining with anti-KSHV LANA. The pictures presented here are representative of multiple immunofluorescence (top panel) and phase contrast (the bottom panel) images of the PBMCs from Cj325-04 at 21 P.I. The scale bars represent 2 µm.

To detect early KSHV infection, a marmoset (Cj325-04) was sacrificed 21 days after intravenous inoculation with 5×10^6^ IU of rKSHV.219. An enzyme-linked immunosorbent assay (ELISA) showed a strong anti-KSHV antibody response (Fig 3A) while PCR products from a variety of tissues to test the presence of KSHV LANA DNA were yielded positive results only for samples from the jejunum and liver (Fig 3B). PBMCs were collected at the time of sacrifice and cultured *in vitro* for one week. Approximately 1/10^4–5^ lymphocytes were GFP-positive and RFP-negative, suggesting latent KSHV infection (Fig 3C). In addition, the KSHV LANA latency-associated protein was readily detected in PBMCs of the rKSHV.219-infected marmoset (Fig 3D). Taken together, these results indicate that KSHV rapidly establishes latent infection in common marmoset PBMCs. It should be noted that the GFP-positive signal from PBMCs of infected marmosets disappeared after an additional week of incubation *in vitro* (data not shown). Due to a low frequency of GFP-positive cells, however, it is unclear whether the GFP-positive cells died or lost the viral genome after a week of incubation *in vitro*. This suggests that while GFP is a convenient marker for viral infection, its use may be limited to the early phase of KSHV infection in marmosets.

### Persistent rKSHV.219 infection is reduced by FK506 treatment

**Figure 4:**
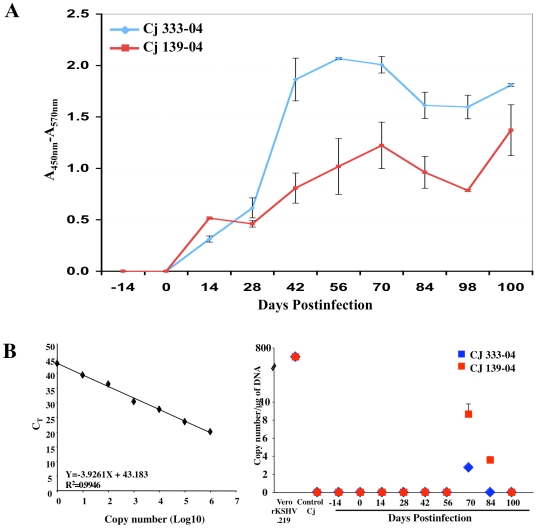
Persistent rKSHV.219 infection is reduced by FK506 treatment. (A) ELISA using the sera (1∶100 dilution) of infected, FK506-treated common marmosets (Cj333-04 and Cj139-04) against the lysed recombinant virus. The results show the averages of two repeats and standard deviations. These assays were reproduced by two independent occasions. (B) Detection of KSHV DNA from FK506-treated, rKSHV.219-infected common marmosets Cj333-04 and Cj139-04. Genomic DNA (100 ng) purified from the PBMCs of FK506-treated marmosets Cj333-04 and Cj139-04 were used for real-time PCR reactions with LANA-specific primers. Genomic DNA from Vero.rKSHV.219 cells (P) was included as a control and the viral DNA copy number/µg of DNA calculated using a standard curve with pcDNA3-LANA DNA. M, 100-bp DNA molecular markers. The results show the averages of two repeats and standard deviations.

**Figure 5:**
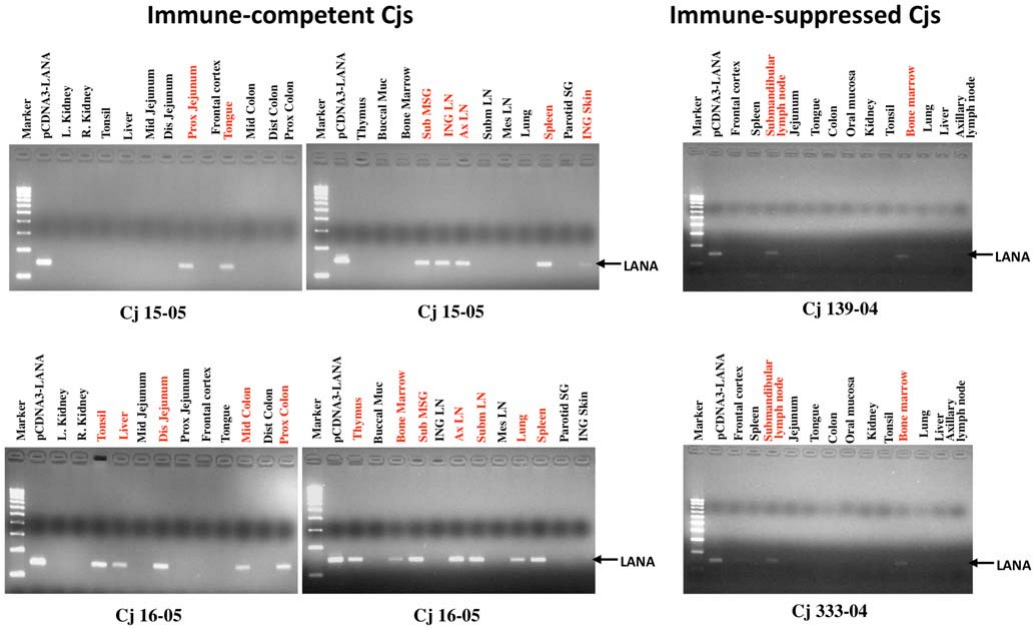
Tissue distribution of viral LANA DNA in rKSHV.219-infected common marmosets. Genomic DNA purified from tissue samples of immune-competent Cj15-05 and Cj16-05 and FK506-treated Cj333-04 and Cj139-04 were used for real-time PCR with LANA-specific primers. Mid, middle; Dis, distal; Prox, proximal; Buccal Muc, buccal mucosa; SubMSG, submandibular salivary gland; ING LN, inguinal lymph node; AxLN, auxiliary lymph node; SubmLN, submandibular lymph node; MesLN, mesenteric lymph node; Parotid SG, parotid salivary gland; ING Skin, inguinal skin. Marker, 100-bp DNA molecular markers. The results show the averages of two repeats and standard deviations.

In transplant recipients, immune suppression is thought to disturb the host's surveillance of KSHV, leading to viral reactivation and an increased systemic viral load [2]. In contrast, rapamycin, an immunosuppressive drug, reduces KSHV-infected PEL cell growth in culture [25] and inhibits the progression of dermal KS in kidney transplant recipients while providing effective immunosuppression [26]. To investigate the direct impact *in vivo* of an immunosuppressive agent on persistent KSHV infection, two common marmosets (Cj333-04 and Cj139-04) were treated with FK506 (Tacrolimus or Fujimycin, 100 µg/kg/day) immunosuppressive drug [27] for 14 days prior to intravenous inoculation with 5×10^6^ IU of rKSHV.219, with treatment continuing for an additional 100 days. Immune-suppressed monkeys inoculated with rKSHV.219 seroconverted to anti-KSHV-positive status within 14 days after inoculation (Fig 4A). Although the anti-KSHV response and KSHV DNA remained present 100 days after infection, they were of a considerably lower magnitude than those of immune-competent monkeys. Compared to untreated animals Cj15-05 and Cj16-05, FK506-treated monkeys Cj333-04 and Cj139-04 showed anti-KSHV antibody responses approximately 2–3 fold lower throughout at 40–80 days postinfection, a shorter duration of time when a positive KSHV LANA DNA signal could be obtained by PCR, and substantially lower copy numbers (Fig 4B). To assess the tissue distribution of rKSHV.219 in infected marmosets, KSHV LANA-specific DNA was amplified from various tissues and organs of immune-competent and immune-suppressed common marmosets. rKSHV.219 DNA was readily detected in numerous tissues of infected monkeys Cj15-05 and Cj16-05, including the tonsils, tongue, lymph nodes, spleen, jejunum, lungs, colon, liver, thymus, submandibular salivary gland, inguinal skin, and bone marrow (Fig 5). In contrast, only the submandibular lymph nodes and bone marrow were positive for rKSHV.219 DNA in FK506-treated animals Cj333-04 and Cj139-04 (Fig 5). These results indicate that immunosuppressive drug treatment leads to a significant reduction of persistent KSHV infection *in vivo*.

### Oral infection of common marmosets with rKSHV.219

**Figure 6:**
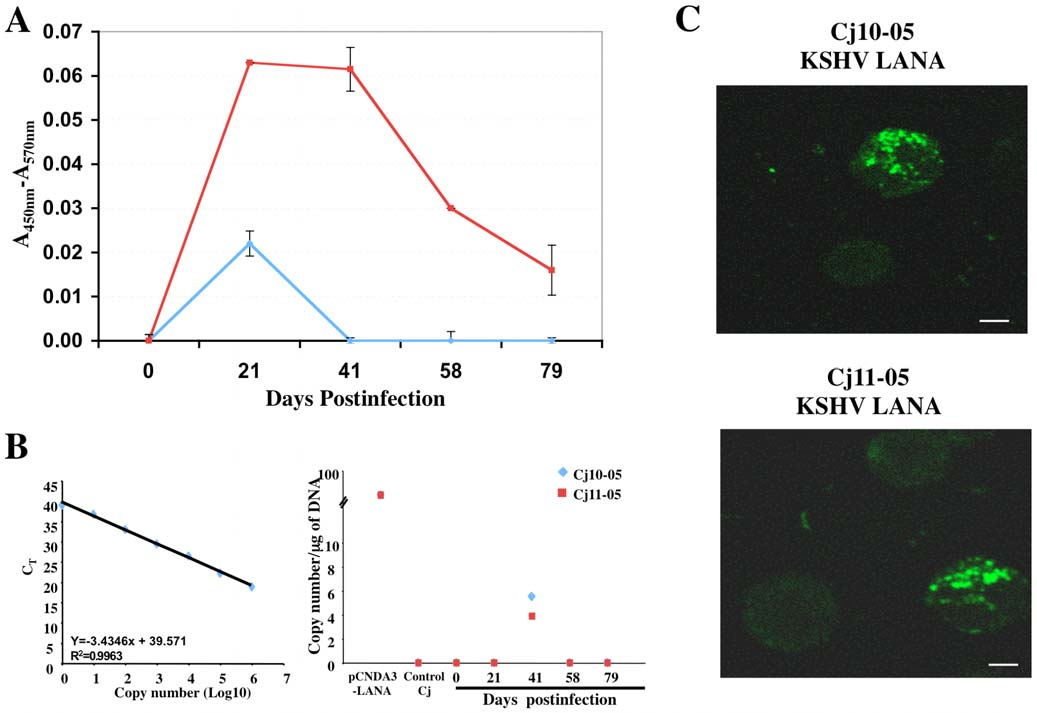
Oral infection of common marmosets with rKSHV.219. (A) Anti-KSHV ELISA using the sera of Cj10-05 and Cj11-05 (1∶5 dilution) was performed as described in Figure 1A. The results show the averages of two repeats and standard deviations. These assays were reproduced by four independent occasions. (B) Real-time PCR of genomic DNA (100 ng) taken from orally infected marmosets Cj10-05 and Cj11-05 using LANA-specific primers at various time points. Purified genomic DNA from Vero.rKSHV.219 cells (P) was included as a positive control. The viral DNA copy number/µg of DNA was calculated using a standard curve with pcDNA3-LANA DNA. The results show the averages of two repeats with standard deviations. (C) Detection of the LANA protein by confocal microscopy. PBMCs from orally infected marmosets Cj10-05 and Cj11-05 were subjected to immunostaining with an anti-LANA antibody, followed by confocal microscopy. The scale bars represent 2 µm.

Two common marmosets were orally inoculated with 5×10^7^ IU of rKSHV.219. Both monkeys inoculated with rKSHV.219 quickly seroconverted to anti-KSHV-positive status within 20 days after inoculation (Fig 6A). However, the anti-KSHV response was much lower in orally infected marmosets than in intravenously infected marmosets and persisted for a shorter period of time (Fig 6A). PBMCs from KSHV-infected marmosets Cj10-05 and Cj11-05 collected on day 41 postinfection were positive for the KSHV LANA sequence but we could not detect KSHV LANA DNA in the PBMCs at any other time under the same conditions (Fig 6B). Like the intravenously infected marmosets, the KSHV LANA protein was also detectable in the PBMCs of orally infected marmosets (Fig 6C). However, since the efficiency of oral infection was much lower than that of intravenous infections, the detection frequency and time period of LANA positivity via confocal microscopy was much lower and more limited with oral infection compared to that in intravenous infection. These results indicate that KSHV infects common marmosets through the oral route but as seen with other viruses [24], oral infection of KSHV is not as efficient as intravenous infection in common marmosets.

**Figure 7:**
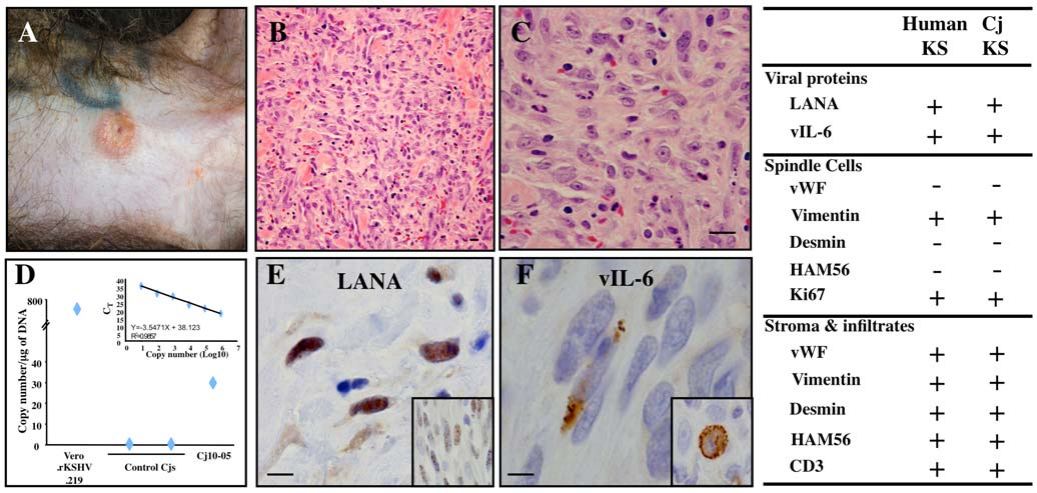
Development of a KS-like skin lesion on an orally infected common marmoset. (A) Skin lesion of rKSHV.219-infected marmoset Cj10-05. (B and C) Histological examination of a marmoset Cj10-05biopsy shows a nonencapsulated dermal mass with characteristics typical of KS lesions, pleomorphic spindle cells along with small blood vessels and extravasated erythrocytes. The spindle cells had a high mitotic index and had infiltrated into the surrounding soft tissues. (D) Real-time PCR of the KS-like skin lesion tissue. Purified genomic DNA (100 ng) from the skin biopsies of two control marmosets and the KS-like lesion of Cj10-05 were used for real time-PCR with LANA-specific primers. Purified genomic DNA from Vero.rKSHV.219 cells was included as a positive control. The viral DNA copy number/µg of DNA was calculated using a standard curve with pcDNA3-LANA DNA. The results show the averages of two repeats with standard deviations. (E and F) Immunohistochemistry of the KS-like skin lesion of Cj10-05 with anti-LANA (E) and anti-vIL-6 (F). The insets in (E) and (F) show the LANA staining of human KS tissue and the vIL-6 staining of KSHV-infected MCD, respectively. The scale bars represent 2 µm (B), 5 µm (C), and 20 µm (E and F). The table on the right is a summary of the results from immunohistochemistry studies of a human KS lesion and the KS-like skin lesion of Cj10-05.

Remarkably, one (Cj10-05) of two animals orally infected with KSHV developed a skin lesion on its ventral abdomen at 41 days postinfection (Fig 7A). This purple skin lesion was approximately 1.5 cm in diameter and had similar histopathological features to those observed in AIDS-associated KS lesions. Histological examination of a biopsy revealed a nonencapsulated dermal mass with characteristics typical of KS lesions. Pleomorphic spindle cells arranged in short bundles were observed, along with small blood vessels and extravasated erythrocytes (Fig 7B and C). The spindle cells had a high mitotic index and had infiltrated into the surrounding soft tissues (Fig 7B and C). Additionally, this KS-like lesion of Cj10-05 was PCR-positive for the KSHV LANA sequence (Fig 7D). Immunohistochemistry with antibodies against LANA, vIL-6, and K8.1 detected KSHV LANA, vIL-6, and K8.1, respectively, in the skin lesion (Fig 7E and F and Supplemental Fig S3). The anti-KSHV LANA, anti-vIL-6, and anti-K8.1 reactivities were specific, and no staining was observed with these antibodies in control skin tissues from naïve common marmosets or in the biopsy tissue of Cj10-05 when the antibodies were replaced with isotype-matched irrelevant antibodies (data not shown and Supplemental Fig S3). Moreover, immunohistochemistry showed virtually identical phenotypes for the skin lesion on Cj10-05 and human KS lesions (table in Fig 7 and Supplemental Fig S4). These results collectively demonstrate that a common marmoset orally infected with KSHV can develop a skin lesion with similar histopathological features to those seen in human KS lesions and is also positive for KSHV DNA and proteins. However, the intensity and prevalence of LANA positive staining within the KS-like lesion of Cj10-05 were not as strong or widespread as those of human KS lesions. In addition, the expression of vIL-6 and K8.1 was restricted to a few cells in the KS-like lesion of Cj10-05, the question of whether these cells were latently infected or lytically replicated requiring further study.

Previous studies have illustrated the persistent infection of rhesus macaque monkeys infected with RRV, a primate homolog of KSHV [15],[28]. Macaques inoculated with RRV alone display transient viremia followed by a vigorous anti-RRV response with no specific clinical features. In contrast, experimental RRV infection of SIV-infected rhesus macaques induces some of the hyperplastic B cell lymphoproliferative diseases which manifest themselves in AIDS patients coinfected with KSHV [15]. Furthermore, cotton-top tamarins (*Saguinus oedipus*) inoculated with human Epstein-Barr virus (EBV) develop diffuse malignant lymphomas resembling human reticulum cell or immunoblastic sarcomas [29],[30]. Additionally, Rhesus lymphocryptovirus, which is very similar to EBV and naturally endemic in rhesus monkeys, can efficiently infect naïve animals orally, with the resulting infection closely mimicking key aspects of human EBV infection [31]. Our study demonstrates that experimental KSHV infection of the common marmoset is highly analogous to its infection of humans, including the means of infection, atypical lymphocytosis, sustained serological responses, latent infection of PBMCs, and virus persistence. However, it should be noted while one of two KSHV-infected marmosets developed KS-like lesion, the number of marmosets used for this experiment is too low to reach the specific conclusion of the frequency of KS-like lesion development induced by KSHV infection. Additional extensive experiments with different conditions such as various KSHV strains and titers and co-infection with HIV-1 or EBV may be able to increase the incidence of KS development. Nevertheless, this model thus provides a unique opportunity to dissect the molecular mechanisms of KSHV infection, persistence, and pathogenesis directly in primates.

We have found that FK506 immunosuppressive drug treatment leads to a significant reduction of persistent KSHV infection *in vivo*. Zoeteweij et al demonstrated that cyclosporine and FK506, specific inhibitors of calcineurin-dependent signal transduction, effectively block the *in vitro* KSHV reactivation induced by ionomycin and thapsigargin, activators of intracellular calcium mobilization [27]. This indicates that FK506 immunosuppressive drug treatment may directly block KSHV reactivation *in vivo* or suppress lymphocyte activation at an early stage of infection, indirectly affecting KSHV persistent infection. Additional experiments, e.g. the timing and type of immune suppression, are necessary to determine the role of host immune competence in the establishment of KSHV persistent infection.

The examination of multiple primate species have demonstrated that γ-herpesviruses are nearly ubiquitous, with homologs of each virus found in a number of different species [32]. Infection of a naïve, natural host by these viruses usually results in a persistent infection that rarely progresses to a pathogenic event outside of specific clinical events. In contrast, cross-species transmission of these viruses can result in profound diseases. For example, herpesvirus saimiri (HVS) is a natural virus of squirrel monkeys, found in over 90% of animals in captivity with no observable pathogenesis [11]. However, transmission of HVS to common marmosets results in a fatal, lymphoproliferative disorder with 100% efficiency. In addition, the transmission of RRV into common marmoset was clear in regard to persistent infection, although it was less conclusive with regards to pathogenicity (unpublished results). Furthermore, viruses could be re-isolated from this animal at multiple time points throughout the experiment (unpublished results). Thus, it is intriguing that the common marmoset is highly susceptible to infection by various pathogens. We speculate that the limited major histocompatibility complex class I (MHC I) polymorphism of common marmosets may contribute to their susceptibility to KSHV infection and pathogenesis. Extensive polymorphism of the MHC is thought to confer immune protection on populations. Restriction fragment length polymorphism analysis showed that there were a limited number of common marmoset MHC class I alleles, whereas the MHC class II gene loci were polymorphic. This may play a role in the susceptibility of this New World primate species to a variety of pathogens. In summary, this is the first animal model that significantly recapitulates the important aspects of KSHV infection in humans and will greatly aid the future designing of anti-viral therapies and be of use in the development of vaccines against KSHV.

## Materials and Methods

### Animals and housing

All common marmosets (*Callithrix jacchus*, Cj) were housed at the New England Primate Research Center (NEPRC) in accordance with the standards of the American Association for Accreditation of Laboratory Animal Care and Harvard Medical School's Internal Animal Care and Use Committee. Common marmosets experimentally inoculated with rKSHV.219 were individually housed in bio-level 3 containment facilities.

### Viral inoculations

Vero cells carrying rKSHV.219 were stimulated with trichostatin A (TSA), the supernatants were harvested to purify rKSHV.219, and the virus titer was determined in 293A and Vero cells by performing a GFP-positive infection assay as described [10]. In order to produce the virus, Vero cells carrying rKSHV.219 were stimulated with 75 nM of TSA for 24 hrs, the media changed, and grown for an additional 48 hrs in DMEM media without FBS. To harvest the virus, the cells were pelleted at 2000 rpm for 10 min with Sorvall SW40 rotor and the ensuing supernatant passed through a 0.45 µm filter, after which it was centrifuged at 18000 rpm for 3 hrs with Sorvall SA-600 rotor to concentrate rKSHV.219. 5×10^6^ IU of this rKSHV.219 was then used to intravenously inoculate the marmosets, for which a flexible, small-bore orogastric tube was utilized to slowly deliver the viral inoculum to the caudal aspect of the oral cavity, tonsils, and nostril mucosa. The inoculum (5×10^7^ IU of rKSHV.219) was dripped slowly over the oral mucosa while the animal was under light sedation, whereby the swallow reflex was maintained. The animals were given ketamine (10–20 mg/kg body weight) intramuscularly prior to blood sampling, inoculation, and euthanasia. The animals underwent periodic blood sampling for viral isolation attempts and the detection of viral DNA. The animals were maintained until the termination of the study or when any of the conditions for euthanasia were met.

### Antibody responses

rKSHV.219 was purified from Vero.rKSHV.219 cells and lysed with a 1% Triton X-100 buffer, after which it was put through five cycles of freezing in liquid nitrogen followed by thawing. The virion proteins were coated onto plates and used to detect reactive antibodies by ELISA as described [12],[28].

### Clinical evaluation, biopsies, and blood samples

Following experimental rKSHV.219 inoculation, all animals were examined daily. Body temperature and clinical data were recorded via an implanted microchip and transponder (Bio Medic Data Systems, Maywood, NJ). PBMCs were obtained prior to inoculation and at various time points throughout the study. Tissues were fixed in 10% neutral-buffered formalin and snap-frozen at −70°C. Blood was obtained at various time points to quantify the viral antibody response, viral load, and complete blood count.

### Histopathology, immunohistochemistry, and confocal microscopy

Formalin-fixed, paraffin-embedded, and snap-frozen tissues were used in immunohistochemical procedures to define the immunophenotype of cells within the skin lesion tissue as described [28]. Briefly, tissue sections were fixed in 2% paraformaldehyde and immunostained with an avidin-biotin-horseradish peroxidase complex technique with diaminobenzidine chromogen. The primary antibodies used in this study were anti-CD20 (B1), anti-CD8 (DK25), anti-CD3 (Nu-Th/1), anti-HLA-DR (CR3/43), anti-Ki67 (MIB-1), anti-vWF (A00082), anti-vimentin (3B4), and anti-HAM56 (M0632). Primary antibodies for viral markers LANA (clone 4A4) and vIL-6 were obtained from Advanced Biotechnologies Inc. (Columbia, MD). Confocal microscopy was used to define the immunophenotypes of virus-infected cells among the PBMCs.

### Detection of rKSHV.219 sequences

To test for the presence of rKSHV.219, DNA was extracted from PBMCs or fresh frozen tissues using a QIAmp tissue kit (Qiagen, Valencia, CA) according to the manufacturer's instructions. DNA was eluted in 50 to 100 µl of sterile water treated with diethylpyrocarbonate and PCR was performed as described below. PBMCs were separated from the whole blood of infected common marmosets using standard Ficoll isolation techniques as described by the manufacturer (Organon Teknika, Malvern, PA).

Real-time PCR was performed with genomic DNA isolated from the PBMCs or tissues of the infected animals and specific primers based on previous analyses of the KSHV sequence [33]. LANA-specific primers [forward primer (5′-CCT CCA TCC CAT CCT GTG TC-3′) and backward primer (5′-GGA CGC ATA GGT GTT GAA GAG-3′)] were used to generate a 146-bp product for LANA detection. ORF9-specific primers [forward primer (5′-ATT CAA GGT CAT ATA CGG CG-3′) and backward primer (5′-CTG GAC AAA ACG ACA GGC TG-3′)] were used to generate a 262-bp product for ORF9 detection. Amplification was performed at 95°C for 25 s and 67.5°C for 60 s for 45 cycles in an iCycler thermal cycler system (Bio-Rad, CA). Data was obtained at *C*_T_ values as per the manufacturer's guidelines (the cycle number at which logarithmic PCR plots cross a calculated threshold line). The PCR products were resolved on a 3% ethidium bromide-stained agarose gel and sequenced to confirm the identities of the KSHV LANA and ORF9 gene fragments.

### Immunoblot analysis

As described in the section “Antibody responses,” virion particles were prepared by stimulating Vero.rKSHV.219 cells with TSA, then by freezing and thawing rKSHV.219 a total of five times in a 1% Triton X-100 buffer using liquid nitrogen. Purified virion proteins (20 µg) were resolved by SDS-polyacrylamide gel electrophoresis (PAGE) and transferred onto a PVDF membrane (Bio-Rad). Immunodetection was achieved with 1∶500 diluted monkey sera. The proteins were visualized by a chemiluminescence reagent (Pierce) and detected by a Fuji chemiluminometer.

### Flow cytometry analysis

5×10^5^ cells per sample were washed with PBS medium containing 1% fetal calf serum and incubated with either fluorescein isothiocyanate-conjugated (FITC) or phycoerythrin-conjugated (PE) monoclonal antibodies for 30 min at 4°C. After washing, each sample was fixed with a 2% paraformaldehyde solution and flow cytometry analysis was performed with a FACS Scan (Becton Dickinson Co.).

## Supporting Information

Figure S1 Immunoblot using the KSHV-infected human sera. KSHV-infected human sera and normal human sera (1∶500 dilution) were used to immunoblot 20 µg of purified virion proteins.
(0.30 MB TIF)

Figure S2 Increase in the CD20+ B cell population in common marmosets infected with rKSHV.219. PBMCs from Cj15-05 (days 210, 320, 380 and 400 P.I.) and Cj16-05 (days 210, 260, 300 and 320 P.I.) were used for flow cytometry analysis with anti-CD3, anti-CD20, and anti-HLA-DR to identify T cells, B cells, and activated lymphocytes, respectively. Uninfected marmosets (Cj190-98, Cj170-04, Cj60-00 and Cj54-05) served as the controls in this experiment. The numbers in the boxes indicate percentages relative to the entire PBMC population.
(1.27 MB TIF)

Figure S3 Immunohistochemistry of the KS-like skin lesion of Cj10-05 with an anti-K8.1 antibody. The inset in the left panel shows an enlarged view of the anti-K8.1 staining of the KS like lesion of Cj10-05. Irrelevant control tissue (right top panel) and KSHV-infected MCD (right bottom panel) were included as negative and positive controls, respectively, for anti-K8.1 staining.
(5.70 MB TIF)

Figure S4 Immunophenotypic comparison of marmoset neoplasm and human KS lesions. Marmoset (A, C, E, G, and I; insert: marmoset positive tissue control) and human tissues (B, D, F, H, and J) were compared immunophenotypically using an ABC immunostaining technique and DAB chromogen for vWF (A, B), vimentin (C, D), desmin (E, F), HAM56 (G, H), and CD3 (I, J). The proliferating spindle cells, stroma, and infiltrating inflammatory cells showed similar immunophenotypic properties. The tumors were composed primarily of vWF-negative, desmin-negative, and vimentin-positive cells supported by a variable stroma containing vWF-positive blood vessels and vimentin-positive cells. Desmin reactivity was observed in the surrounding tissues. Both tumors were infiltrated by HAM56-positive macrophages and significant numbers of CD3-positive lymphocytes. Spindleoid cells were uniformly negative for these markers.
(8.07 MB TIF)
