# Supplementary figures and images for: Non-Human Primate Model of Kaposi's Sarcoma-Associated Herpesvirus Infection
Source: PLoS Pathog. 2009 Oct 2;5(10):e1000606. doi: 10.1371/journal.ppat.1000606 (PMC2745662; doi:10.1371/journal.ppat.1000606)

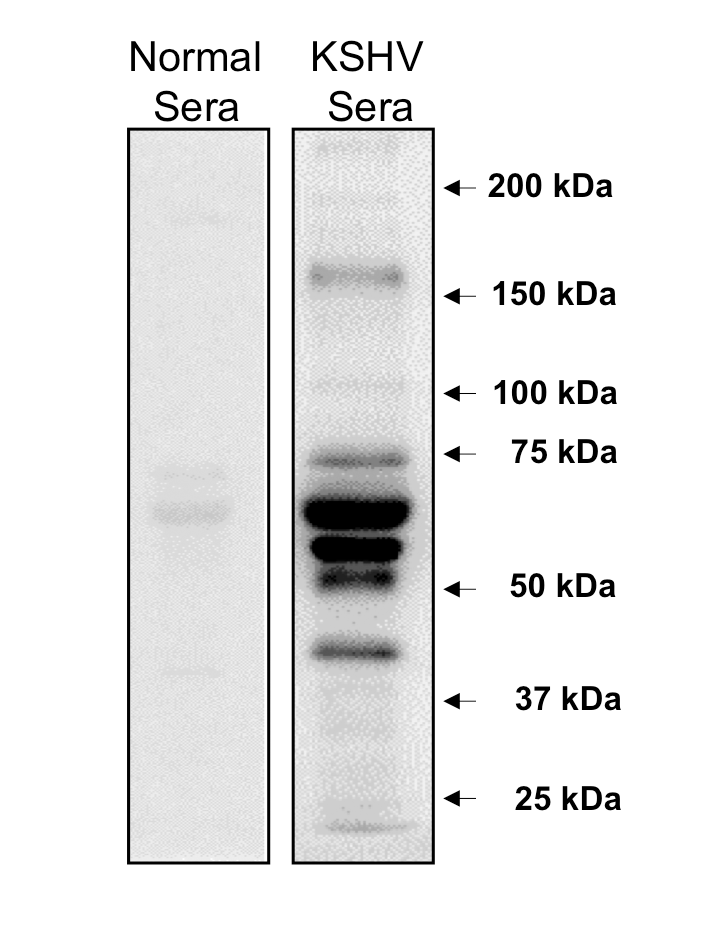

Supplement: Figure S1 — Immunoblot using the KSHV-infected human sera. KSHV-infected human sera and normal human sera (1∶500 dilution) were used to immunoblot 20 µg of purified virion proteins. (0.30 MB TIF) [file ppat.1000606.s001.tif]

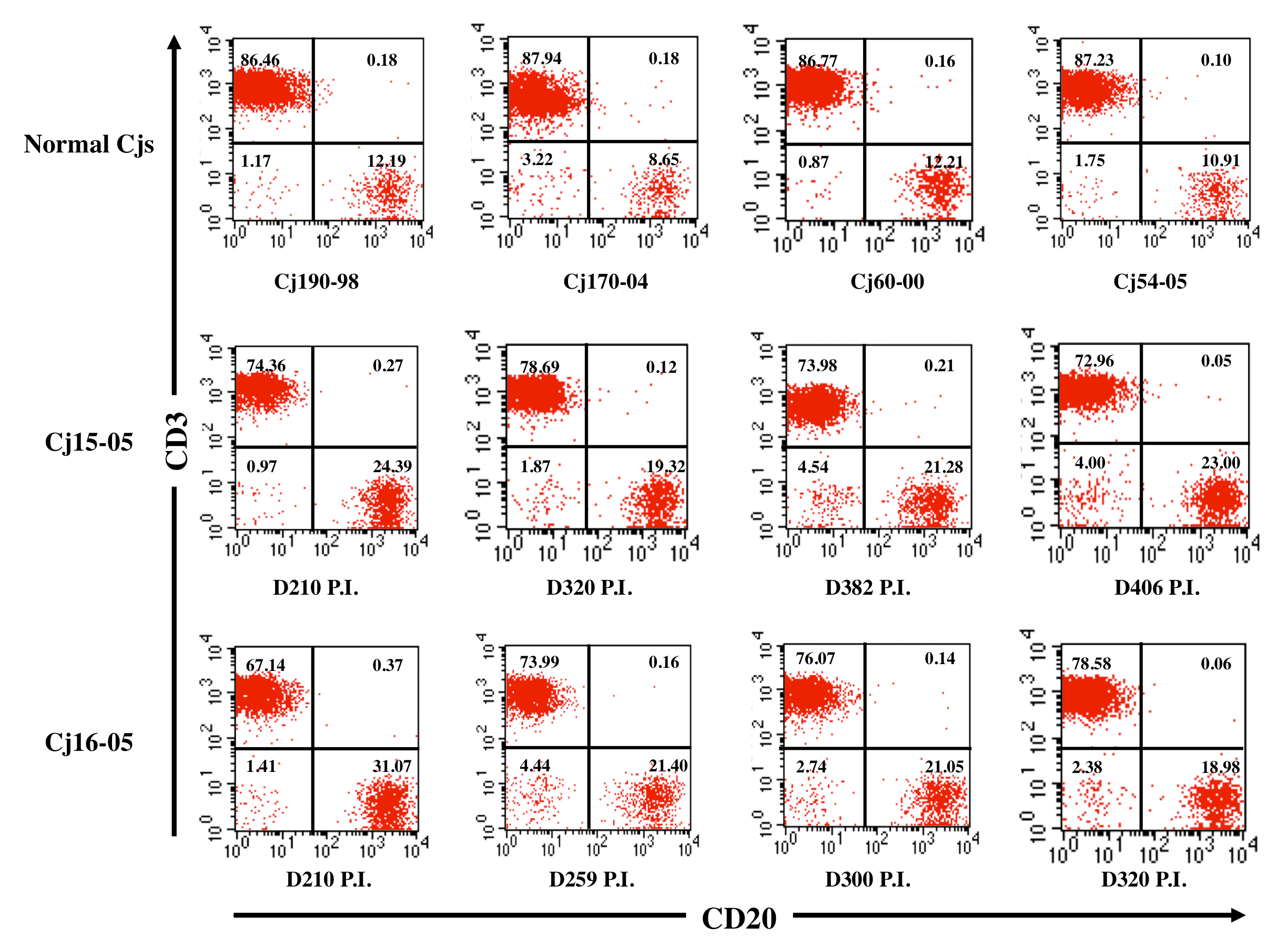

Supplement: Figure S2 — Increase in the CD20+ B cell population in common marmosets infected with rKSHV.219. PBMCs from Cj15-05 (days 210, 320, 380 and 400 P.I.) and Cj16-05 (days 210, 260, 300 and 320 P.I.) were used for flow cytometry analysis with anti-CD3, anti-CD20, and anti-HLA-DR to identify T cells, B cells, and activated lymphocytes, respectively. Uninfected marmosets (Cj190-98, Cj170-04, Cj60-00 and Cj54-05) served as the controls in this experiment. The numbers in the boxes indicate percentages relative to the entire PBMC population. (1.27 MB TIF) [file ppat.1000606.s002.tif]

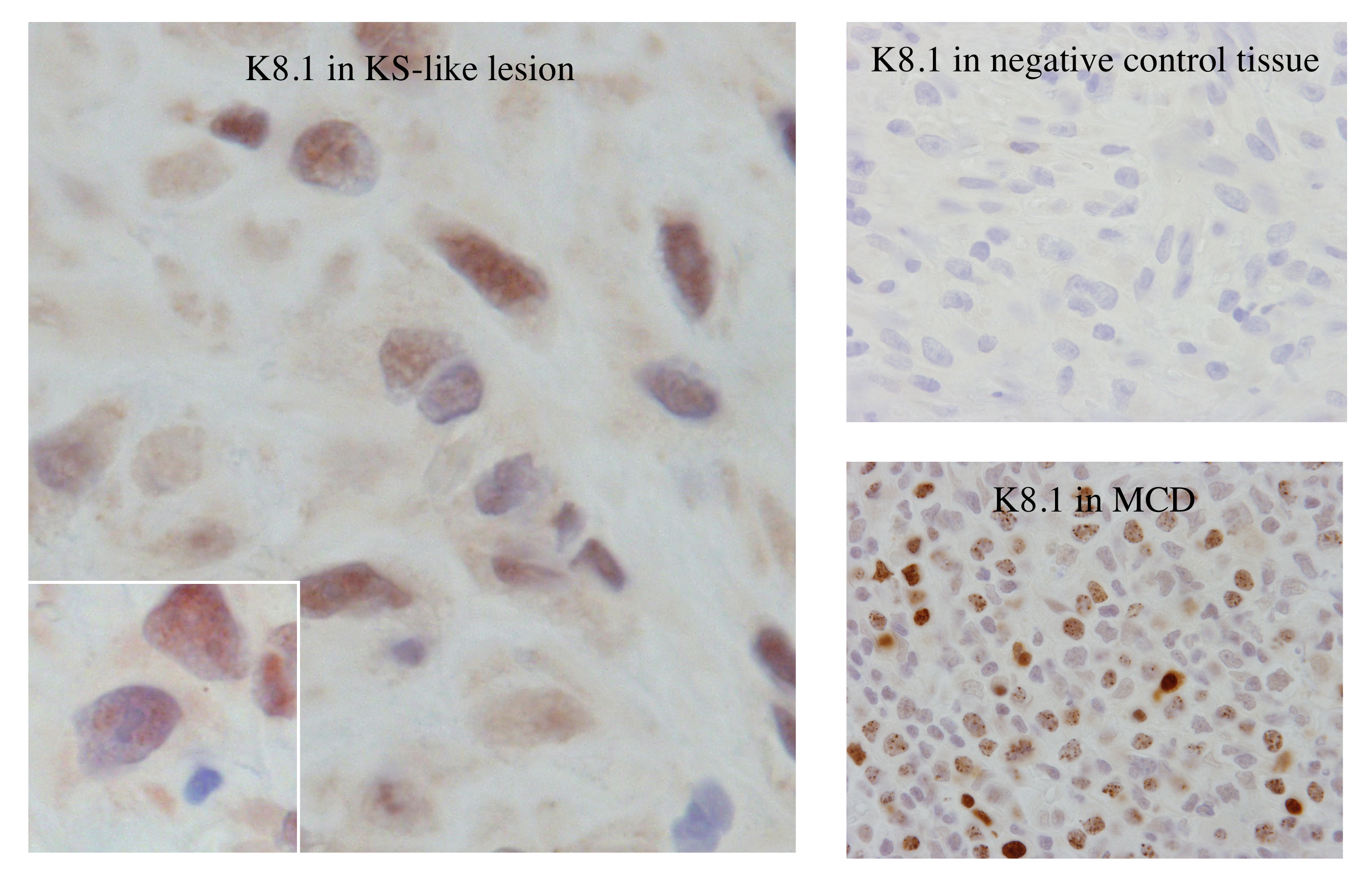

Supplement: Figure S3 — Immunohistochemistry of the KS-like skin lesion of Cj10-05 with an anti-K8.1 antibody. The inset in the left panel shows an enlarged view of the anti-K8.1 staining of the KS like lesion of Cj10-05. Irrelevant control tissue (right top panel) and KSHV-infected MCD (right bottom panel) were included as negative and positive controls, respectively, for anti-K8.1 staining. (5.70 MB TIF) [file ppat.1000606.s003.tif]

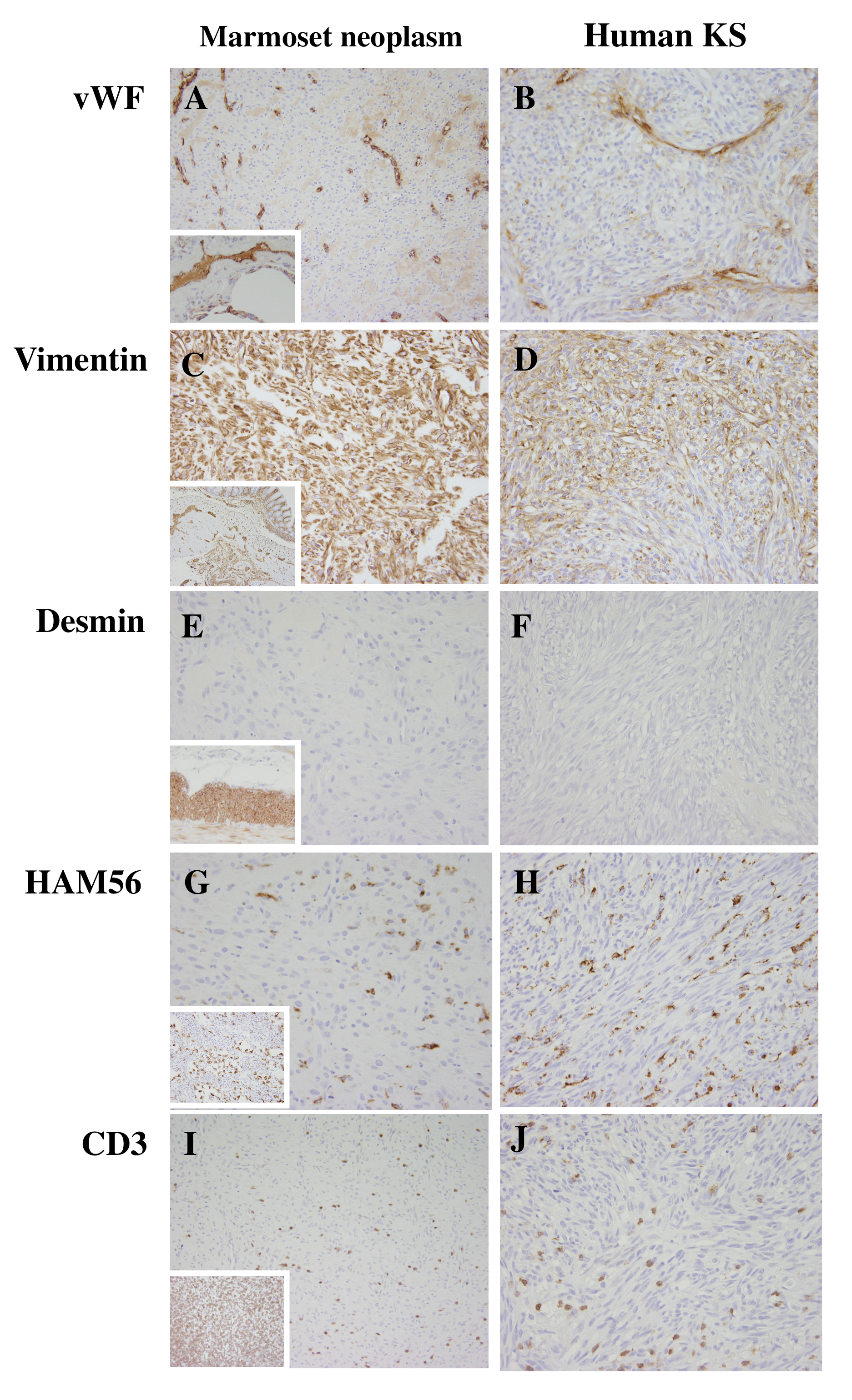

Supplement: Figure S4 — Immunophenotypic comparison of marmoset neoplasm and human KS lesions. Marmoset (A, C, E, G, and I; insert: marmoset positive tissue control) and human tissues (B, D, F, H, and J) were compared immunophenotypically using an ABC immunostaining technique and DAB chromogen for vWF (A, B), vimentin (C, D), desmin (E, F), HAM56 (G, H), and CD3 (I, J). The proliferating spindle cells, stroma, and infiltrating inflammatory cells showed similar immunophenotypic properties. The tumors were composed primarily of vWF-negative, desmin-negative, and vimentin-positive cells supported by a variable stroma containing vWF-positive blood vessels and vimentin-positive cells. Desmin reactivity was observed in the surrounding tissues. Both tumors were infiltrated by HAM56-positive macrophages and significant numbers of CD3-positive lymphocytes. Spindleoid cells were uniformly negative for these markers. (8.07 MB TIF) [file ppat.1000606.s004.tif]
